# Widening Disparities in Teen HPV Vaccinations during COVID-19 Pandemic: A Case Study from Veneto Region (Italy)

**DOI:** 10.3390/vaccines10122120

**Published:** 2022-12-11

**Authors:** Luca Perin, Alessandra Dal Zotto, Marta Savio, Antonio Stano, Lorenzo Bulegato, Luca Tribbia, Roberta Donà, Matilde Tomasi, Silvia Fietta, Antonio Ferro, Vincenzo Baldo, Mario Saugo, Silvia Cocchio

**Affiliations:** 1 Department of Prevention of Local Health Unit n. 7, Veneto Region, 31011 Venice, Italy; 2 Post-Graduate School of Hygiene and Preventive Medicine, Department of Environmental and Prevention Sciences, University of Ferrara, 44121 Ferrara, Italy; 3 Italian Society of Hygiene, Preventive Medicine and Public Health, 10126 Torino, Italy; 4 Department of Cardiac, Thoracic, Vascular Sciences and Public Health, University of Padua, 35122 Padova, Italy

**Keywords:** HPV, vaccination coverage, adolescent

## Abstract

Introduction: In Local Health Unit 7, human papilloma virus (HPV) vaccination campaigns for 12-year-olds have long been implemented by the vaccination services of the Department of Prevention. Due to the pressure of the COVID-19 pandemic on these services, an emergency vaccination campaign was directly managed by primary care pediatricians (PCPs). An initial evaluation of this experience was conducted. Materials and methods: Data on 12-year-olds assisted by PCPs belonging to the 2006 (pre-pandemic) and 2008 (pandemic) birth cohorts were extracted, along with HPV vaccination data. Health district, gender, citizenship, socioeconomic status, and PCPs were evaluated as possible influencing factors in a two-level logistic regression (second level: single PCP). Results: The HPV vaccination gap between males and females increased significantly for the 2008 birth cohort compared to the 2006 birth cohort (11 vs. 4 percentage points). As for PCPs, the vaccination uptake range was 4–71% for the 2008 birth cohort vs. 32–85% for the 2006 cohort. The proportion of variance explained at the second level was overall equal to 9.7% for the 2008 cohort vs. 3.6% for the 2006 cohort. Conclusions: The vaccination campaign carried out during the peak of the COVID-19 pandemic increased the HPV vaccination gaps among Health Districts, genders, and individual PCPs, probably due to a lack of homogeneity in professional practices and attitudes toward HPV vaccination. Catch-up interventions are required in the immediate term, while an equity-lens approach should be taken for reprogramming the vaccination campaign. Greater involvement of schools and families could ensure a more equitable approach and a better uptake.

## 1. Introduction

The COVID-19 pandemic has dramatically disrupted the delivery of routine vaccines for children and adolescents. At the beginning of the pandemic, a temporary suspension of vaccination services was enforced, while the fear of contagion limited the attendance at these services. When the COVID-19 vaccines were finally available, a large proportion of the vaccination staff were reallocated to COVID-19 vaccination centers [1,2,3].

In Italy, the human papilloma virus (HPV) vaccination, unlike most pediatric immunizations, is not mandatory, resulting in a striking reduction in coverage, with the first HPV dose of 20 percentage points for 12-year-old girls and 16 percentage points for 12-year-old boys [4].

Even before the pandemic, inequalities regarding access to HPV vaccinations for adolescents were described in many countries worldwide. Lower vaccination coverage has been found among ethnic minorities and low-educated or socially disadvantaged population groups; these disparities are less marked when the vaccination is offered for free, as is the case in Italy [5,6]. Another important inequality is related to gender: vaccination coverage is lower in boys than in girls (for example, in Italy, the vaccination coverage for one HPV dose in the 2008 cohort was 41.3 vs. 50.1%) [4]. This can mainly be attributed to cultural factors: parental decisions regarding vaccines are predominantly shaped by the perceived benefits of the vaccine. Mothers and fathers generally fear that their unvaccinated daughters will develop cervical cancer; however, acuminate condyloma, penile, anal, and head–neck cancer are also associated with HPV infections in boys [7]. Parents’ perceptions are also influenced by the national health policy: in Italy, the vaccination against HPV was offered to 12-year-old girls in 2007 and to 12-year-old boys only 7 years later [4].

Healthcare professionals, particularly general practitioners (GPs) and primary care pediatricians (PCPs), can have a significant influence on the HPV vaccination coverage of their patients [8]. Physicians’ recommendations have the greatest influence on the parents’ acceptance, and medical counseling is closely linked to the health professionals’ knowledge, attitudes, and abilities [9,10,11,12,13]. For example, in the United States, up to one-third of PCPs and GPs reported that they anticipated uncomfortable conversations if they recommended HPV vaccinations for 11- and 12-year-olds [11,12]. 

The COVID-19 emergency seriously tested and reshaped the vaccination services [1,14,15,16,17]. Previous teen HPV vaccination campaigns were interrupted, leaving space for an eventual increase in disparities in vaccination uptake. This case study examined the HPV vaccination strategies of Local Health Unit 7 Pedemontana (LHU 7) in the Veneto region, Italy. Among cohorts of 12-year-olds, those born up to the year 2007 were vaccinated by the vaccination services of the Department of Prevention, and those born in 2008 were vaccinated directly by PCPs, following an agreement signed in July 2021. Both cohorts were vaccinated with GARDASIL 9^®^. The purpose of this study was to assess the impact of this change on the HPV vaccination coverage in terms of the disparities in access by gender, citizenship, and socioeconomic status, and among different PCPs. 

## 2. Materials and Methods

The study population included birth cohorts from 2006 (pre-pandemic period) and 2008 (pandemic period). Adolescents were linked to their primary care physician on 1 January 2019 (2006 cohort) or 30 April 2022 (2008 cohort) using the population register; those assisted by a GP were not included. 

Those in the 2006 cohort received personal invitations by mail with the date, time, and place of vaccination from the vaccination services of the Department of Prevention, while those in the 2008 cohort were referred to their respective PCP for vaccination. Each adolescent in the study was characterized by gender (male/female), citizenship (Italian/foreign), and socioeconomic status at the municipal level (Italian deprivation index) [18]. Data related to the administration of the first HPV dose were extracted from the vaccination register of the Veneto region (1 January 2019 to 31 December 2019 for the 2006 cohort and 1 July 2021 to 30 April 2022 for the 2008 cohort). For the 2008 cohort, SARS-CoV-2 infections and COVID-19 vaccinations during the same observation window were also considered, to assess how they influenced the access to HPV vaccinations. The outcome was receiving the first dose of the HPV vaccination within the time windows described above.

The impact on vaccination coverage disparities by gender, citizenship, and socioeconomic status was measured in terms of the relative risk ratio, using a generalized linear model for the binomial family, considering the risk ratio measured in the 2006 cohort as a reference. A multilevel and multivariate logistic model with random effects was used to take into account the hierarchical structure of the data, with the second level of aggregation being PCPs. The variability among PCPs was measured using the intraclass ρ correlation index, which describes the percentage of variance explained at the second level. To compare the variability among PCPs in the two cohorts under study, the median odds ratio (MOR) was calculated using a generalized structural equation model (GSEM). MOR can be conceptualized as the median increased risk if another PCP with higher HPV coverage was chosen. Therefore, MOR is equal to 1 if there is no coverage difference between PCPs, and the higher the value, the greater the difference [19]. This approach was used to evaluate the improvement in flu coverage for at-risk groups following the General Service Contract of GPs in the UK [20]. Stata 17^®^ software was used for statistical analysis. 

## 3. Results

A total of 2754 12-year-old patients were enrolled in the 2006 cohort (vaccinated in the pre-pandemic period by the vaccination services of the Prevention Department), and 2208 in the 2008 cohort (vaccinated in the pandemic period by PCPs). As shown in Table 1, the demographic and socioeconomic factors of the two cohorts were similar; there was only a slightly higher proportion of 12-year-olds with North African citizenship in the 2008 cohort.

As shown in Table 2, 1986 adolescents in the 2006 cohort (72.1%) and 1281 in the 2008 cohort (58.0%) were vaccinated with the first HPV dose. The male vs. female differential was 5 percentage points for the 2006 cohort and 18 percentage points for the 2008 cohort, and it was more pronounced in Health District A (HD A). In relative terms, the reduction in coverage (relative risk ratio), to the disadvantage of males, increased by 14% (Table 2).

**Figure 1:**
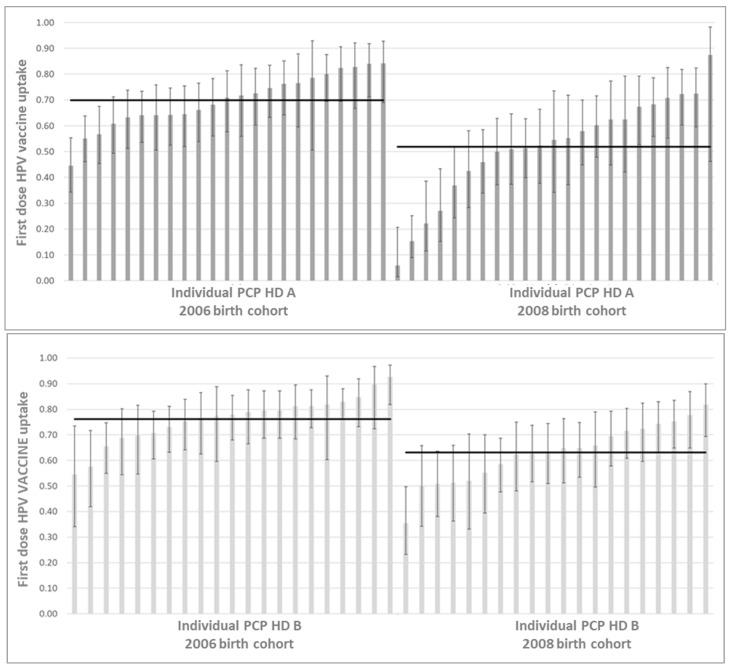
First dose of HPV vaccine among 12-year-olds in 2006 and 2008 birth cohorts, assisted by primary care pediatricians (PCPs). Health Districts (HD) of Local Health Unit 7, Veneto Region. Whiskers: 95% confidence interval for the estimated mean.

Foreign citizenship influenced the vaccination coverage significantly and homogeneously in both birth cohorts (2006 cohort: RR 0.83; 95% CI 0.78–0.89; 2008 cohort: RR 0.87; 95% CI 0.78–0.98), but there was no evidence of a differential impact (RRR: 0.96; 95% CI: 0.84–1.09). The municipal index of deprivation of the 52 municipalities of LHU 7 did not show any effect (2006 cohort: RR intertertile 1.05; 95% CI 0.99–1.12; 2008 cohort: RR intertertile 0.96; 95% CI 0.88–1.04). The range of teen vaccination coverage by individual PCPs showed an increase for the 2008 birth cohort compared to the 2006 cohort (HD A 6–88% vs. 45–84%; HD B 35–82% vs. 55–93%) (Figure 1).

The multilevel logistic model with mixed effects, adjusted for sex and citizenship, shows that the intraclass ρ correlation coefficient (between PCP variance, adjusted for gender and citizenship) increased substantially in LHU 7 (12.5% in the 2008 cohort vs. 5.1% in the 2006 cohort) and in Health District A (16.7% vs. 3.8%), while it remained substantially unchanged in HD B (4.4% vs. 3.2%).

In the 2008 cohort, COVID-19 vaccinations within the observation window (July 2021–April 2022) were associated with a significant increase in HPV coverage (34.0% in unvaccinated vs. 68.1% in vaccinated adolescents; Chi^2^ = 5.15, df = 1, *p* = 0.023), whereas the SARS-CoV-2 infection had the opposite effect (64.1% for those who were not infected vs. 47.7% for those who were; Chi^2^ = 143.01, df = 1, *p* = 0.000). Of note, these two factors have a similar distribution among PCPs; therefore, the intraclass correlation coefficient does not change (ρ = 1%, 95% CI 8–20% in the model adjusted for gender and citizenship and *p* = 12%, 95% CI 9–19% in the model including the COVID-19 vaccination and SARS-CoV-2 infection).

**Figure 2:**
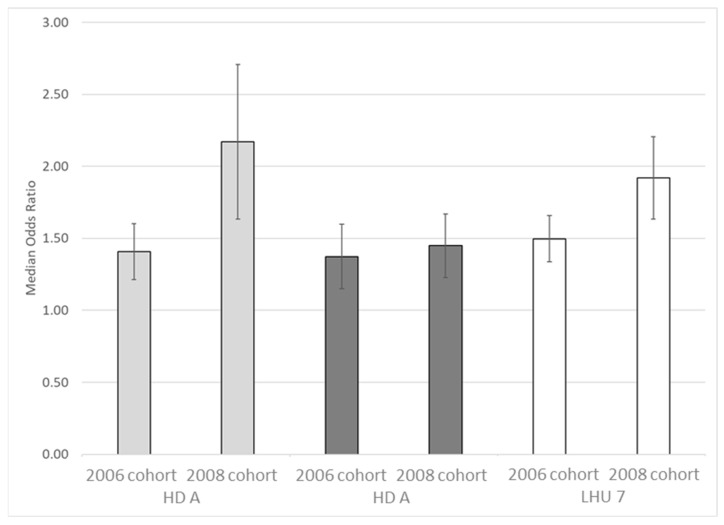
Median odds ratios for first dose of HPV vaccine among 12-year-olds in 2006 and 2008 birth cohorts assisted by primary care pediatricians, adjusted for gender and citizenship. Health Districts of Local Health Unit 7, Veneto region.

Finally, the variability attributable to PCPs in the two birth cohorts was measured quantitatively, using median odds ratios and relative confidence intervals (Figure 2).

The median odds ratio increased significantly in HD A (MOR = 2.17, 95% CI 1.63–2.71 for 2008 cohort vs. MOR = 1.41, 95% CI 1.21–1.60 for 2006 cohort; Chi^2^ = 4.84, df = 1, *p* = 0.028), whereas it remained almost unchanged in HD B (MOR = 1.45; 95% CI 1.23–1.67 for 2008 cohort vs. MOR = 1.37, 95% CI 1.15–1.60 for 2006 cohort; Chi^2^ = 0.16, df = 1, *p* = 0.690). Overall, in LHU 7, the increase in coverage variability between PCPs was significant (MOR = 1.92, 95% CI 1.63–2.21 vs. MOR = 1.50, 95% CI 1.34–1.66; Chi^2^ = 4.52, df = 1, *p* = 0.034).

## 4. Discussion

A field experiment carried out during the COVID-19 pandemic shows that referring 12-year-olds to PCPs for their HPV vaccinations resulted in an increase in vaccination coverage disparities by gender (to the disadvantage of males) and among PCPs. This result is more evident in Health District A, which already showed lower coverage in the 2006 cohort.

Other studies have evaluated the impact of the COVID-19 pandemic in terms of the inequality of access to routine vaccinations for children and adolescents who belong to ethnic minorities or live in rural communities or deprived neighborhoods 1.14–17.21. In this study, no disparity in vaccination coverage was observed among non-Italian adolescents, who represent about 7% of the birth cohorts and are distributed uniformly within LHU 7. In addition, no reduction in vaccination coverage was found among adolescents living in disadvantaged municipalities (more detailed neighborhood data are unavailable) [14].

A lower HPV vaccine coverage among male adolescents has long been observed worldwide and is consistent with the delayed recommendation, or lack of recommendation, for HPV vaccinations for boys. The reduction in vaccination coverage in the males from the 2008 cohort compared to the females is an original finding of this study, and may be reasonably linked to the educational attitudes of different PCPs. Vaccinating a 12-year-old boy for HPV may require a conversation with the parents to address objections such as, “My son is too young and not yet sexually active”, or, “HPV infection affects young and adult women”. Parental health literacy is important, but medical counselling can make a difference [21,22]. Female PCPs and those from ethnic minorities generally show a more proactive attitude toward HPV counseling, and numerous intervention studies have shown that the educational aptitude and skills of PCPs can be effectively improved [12,23,24,25,26].

The novelty of this study is the formal assessment of the disparity in HPV vaccination coverage observed among primary care pediatricians, before and after the COVID-19 pandemic. The widening of vaccination coverage disparities among PCPs for the 2008 cohort suggests the presence of different educational attitudes and capabilities, as well as different management methods adopted by PCPs, such as providing vaccination appointments and access times, making it easy to schedule and change an appointment (through an office secretary or self-booking computer-assisted system), and active calling (sending an invitation with the date and time of the appointment).

PCPs who work alone cannot delegate to other professionals activities that do not require specific medical skills (consultations, vaccine stock management, control of vaccine expiration, inoculation, and post-vaccination surveillance) [10]. This may help explain the observed gap between the two HDs, given that in HD B, pediatric group practices are historically more developed and consolidated. In the UK vaccination experience, such technical, organizational, and managerial aspects are codified in the standard primary care agreement [27].

HPV vaccination coverage in the 2008 cohort was influenced by the SARS-CoV-2 infection and the COVID-19 vaccination. The former may have reduced vaccination coverage because of the isolation measures that required postponing the appointment. In contrast, the COVID-19 vaccination increased the odds of receiving an HPV vaccination, which could have resulted from a greater propensity toward vaccinations. Interestingly, these factors were distributed homogeneously in the target population and did not significantly influence the large gap observed among PCPs.

This local experience shows that careful monitoring of the disparities in HPV vaccination coverage can offer important insights for the evaluation of HPV vaccination campaigns and programs. 

## 5. Conclusions

After several COVID-19 pandemic waves, it is time for resilience and recovery, and this is also true for teen HPV vaccinations. First, it is necessary to reverse the sharp drop in coverage with catch-up campaigns, to avoid an undue burden of HPV infection and disease in the youth population in the medium term [28]. 

Many strategies have been shown to be effective in improving HPV vaccination coverage among adolescents. For example, social marketing campaigns and personalized invitations and reminders are effective at minimizing socioeconomic variability in the uptake of routine HPV immunizations [29,30]. Important partners are schoolteachers, who can offer an educational approach and universal access, as demonstrated by the leading experience of the Scottish vaccination program, which was also followed in Europe by the UK, Belgium, Sweden, and Norway [31,32,33,34]. School programs have influenced both parents and pre-adolescents in terms of their knowledge, attitudes, and willingness to vaccinate in Italy as well [35,36]. 

In the actual Venetian context, many of the proposed interventions are reasonably easier to implement within the framework of the vaccination services of the Department of Prevention than among PCPs. Whatever the health policy indication, the “health equity lens” can provide invaluable help in recognizing and reducing inequalities, and identifying the most effective strategies for reprogramming [6,37].

## Figures and Tables

**Table 1:** Main characteristics of study population (12-year-olds born in 2006 and 2008 and assisted by a primary care physician, Local Health Unit 7, Veneto region).

		2006 Cohort, Vaccinated by Dept. of Prevention		2008 Cohort, Vaccinated by PCPs (Jul 2021–April 2022)		Chi^2^	
		N	%	N	%	Value; df	*p*
Gender	Female	1268	46.0%	1066	48.3%	2.46; 1 df	0.117
	Male	1486	54.0%	1142	51.7%		
Citizenship	Italian	205	7.4%	159	7.2%	0.11; 1 df	0.745
	Foreign	2549	92.6%	2049	92.8%		
North African	No	2735	99.3%	2155	97.6%	25.1; 1 df	0.000
	Yes	19	0.7%	53	2.4%		
Municipal deprivation index	1st tertile	894	32.5%	721	32.7%	4.54; 3 df	0.209
	2nd tertile	1009	36.6%	792	35.9%		
	3rd tertile	756	27.5%	639	28.9%		
	Missing	95	3.4%	56	2.5%		
Vaccinated for COVID-19 (Jul 2021–April 2022)	No	-	-	653	29.6%	-	-
	Yes	-	-	1555	70.4%		
COVID-19 positive (Jul 2021–April 2022)	No	-	-	1394	63.1%	-	-
	Yes	-	-	814	36.9%		

**Table 2:** First dose of HPV among 12-year-old boys and girls born in 2006 and 2008 assisted by a primary care pediatrician, Local Health Unit 7, Veneto region.

		Males		Female		Total		RR Males vs. Females			2008 vs. 2006 Cohort		
		N	%	N	%	N	%	RR	IC 95%		RRR	IC 95%	
HD A	2006 Cohort	471	65%	419	69%	890	67%	0.93	0.87	1.01	**0.85**	**0.73**	**0.98**
	2008 Cohort	219	44%	253	57%	472	50%	**0.77**	**0.68**	**0.88**			
HD B	2006 Cohort	577	76%	519	78%	1096	77%	0.97	0.92	1.03	**0.87**	**0.78**	**0.96**
	2008 Cohort	380	59%	429	69%	809	64%	**0.86**	**0.79**	**0.93**			
LHU 7	2006 Cohort	1048	71%	938	74%	1986	72%	**0.95**	**0.91**	**1.00**	**0.86**	**0.79**	**0.94**
	2008 Cohort	599	52%	682	64%	1281	58%	**0.82**	**0.76**	**0.88**			

Bold text indicates a *p*-value < 0.0.5

## Data Availability

The data supporting the findings of this study are available from the corresponding author upon reasonable request, and first has to be approved by Local Health Unit 7 Pedemontana (LHU 7) of the Veneto region.
